# An Improvement of Robot Stiffness-Adaptive Skill Primitive Generalization Using the Surface Electromyography in Human–Robot Collaboration

**DOI:** 10.3389/fnins.2021.694914

**Published:** 2021-09-14

**Authors:** Yuan Guan, Ning Wang, Chenguang Yang

**Affiliations:** Bristol Robotics Laboratory, University of the West of England, Bristol, United Kingdom

**Keywords:** learning from demonstration, human-robot collaboration, Imitation learning, surface electromyography signal, human-like stiffness adaptation, action recognition, robot skill generalization, decision-making

## Abstract

Learning from Demonstration in robotics has proved its efficiency in robot skill learning. The generalization goals of most skill expression models in real scenarios are specified by humans or associated with other perceptual data. Our proposed framework using the Probabilistic Movement Primitives (ProMPs) modeling to resolve the shortcomings of the previous research works; the coupling between stiffness and motion is inherently established in a single model. Such a framework can request a small amount of incomplete observation data to infer the entire skill primitive. It can be used as an intuitive generalization command sending tool to achieve collaboration between humans and robots with human-like stiffness modulation strategies on either side. Experiments (human–robot hand-over, object matching, pick-and-place) were conducted to prove the effectiveness of the work. Myo armband and Leap motion camera are used as surface electromyography (sEMG) signal and motion capture sensors respective in the experiments. Also, the experiments show that the proposed framework strengthened the ability to distinguish actions with similar movements under observation noise by introducing the sEMG signal into the ProMP model. The usage of the mixture model brings possibilities in achieving automation of multiple collaborative tasks.

## 1. Introduction

According to current trends, robots are more applicable in factories, medical, social service, and other domains and will become more extensive. More and more industries consider or have established complete autonomous robot systems or human–robot collaboration platforms to replace human labor entirely with machines or assist people in their work. This benefited from the development of robotics, communication, and artificial intelligence technologies, which indicates that robots will considerably liberate part of the labor in high-repetition, high-fatigue works. It provides services autonomously in more complex work scenarios and may require collaborating with multiple agents, such as the assembly of 3C products, robot-assisted surgery, and physical and social assistance. Typically, collaborative scenes that involve multiple agents tend to have relatively complex environmental conditions and great diversity of tasks (Villani et al., 2018). Only by improving the accuracy of robot decision making and proffering it good adaptability and safety can it meet the ever-increasing demand in the future. Robot Imitation Learning [i.e., Learning from Demonstration (LfD)] dramatically improved robot pre-programming efficiency (Argall et al., 2009). People are transferring knowledge to the robot by endowing robots the ability to imitate via human demonstrations. This is a more intuitive and convenient way of teaching/programming. Because LfD modularizes skills, it simplifies the re-programming process when switching between work content and scenes. It does not require people with expertise in robots and computing to design task-dedicated programs.

In the previous literature, the demonstration-based robot skill learning framework usually comprises the following three processes: (1) human skill demonstrations; (2) skill mathematical expression and modeling; (3) skill reproduction and generalization. While demonstrating, the demonstrator selects the appropriate demonstration interface. Under typical circumstances, the interfaces are divided into three principal categories: kinesthetic teaching, teleoperation, and passive observation. Different interfaces have their advantages and limitations (Billard et al., 2016). The experiments conducted in this article employ the teleoperation method. Demonstrator using this method usually only pays attention to the movement of the robot end-effector and ignores the preceding joints. Nevertheless, because the movement of the demonstrator is less physically restricted, which makes it more flexible. This article presents a novel framework that improves the skill generalization efficiency and accuracy, and we exploit the benefits from bioelectrical signals like electromyography to better infer human intents and transfer human stiffness regulation strategy to the robot, which highly relates to processes 2 and 3 mentioned above.

**Figure 1:**
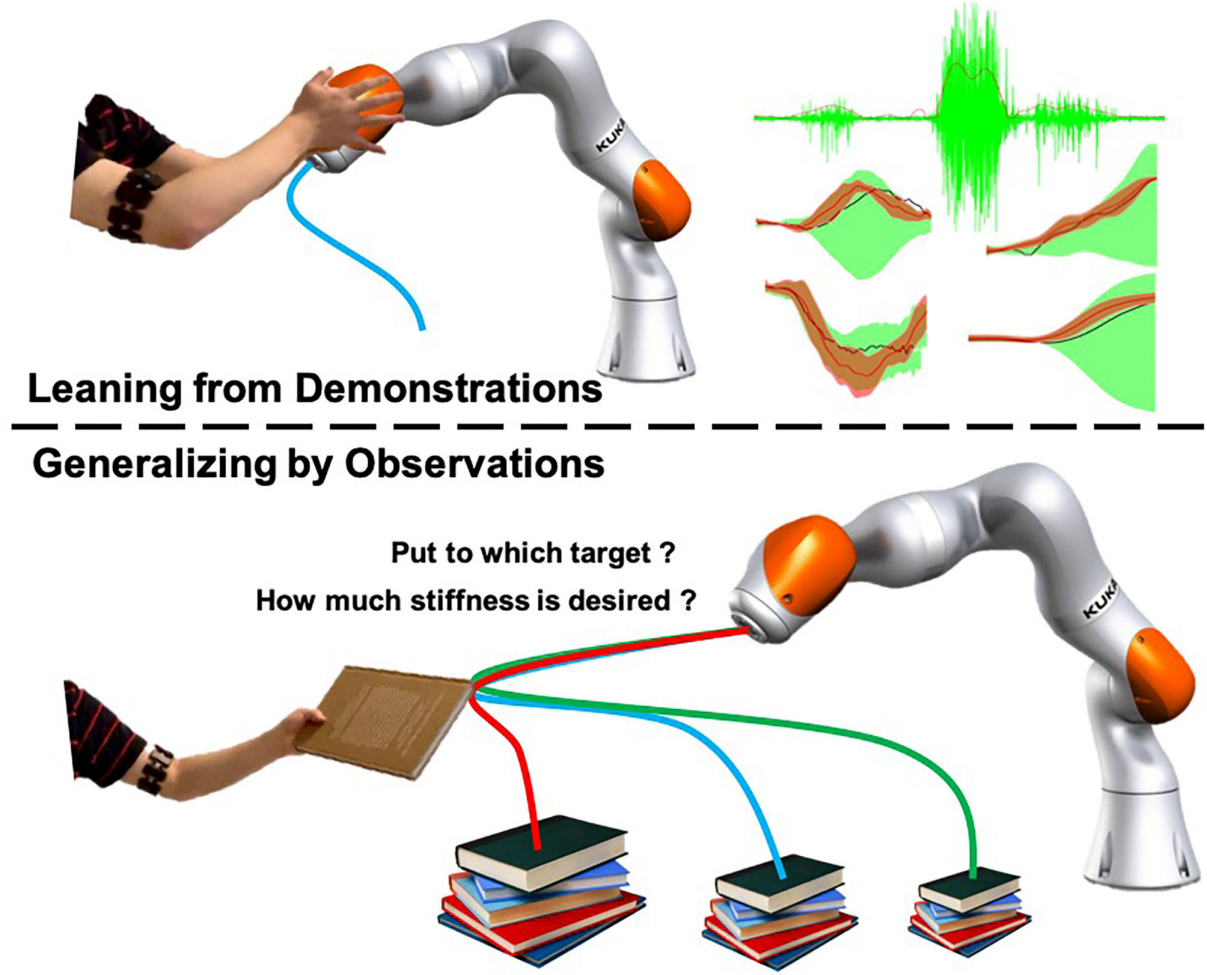
An illustrative figure of the proposed framework. People demonstrate motion and stiffness adaptation skills for both human and robot and encode the collaborative skill using a high-dimensional Probabilistic Movement Primitives (ProMPs) model. The robot will be able to generates appropriate motion and stiffness by observing incomplete data from human.

Stiffness is critical in robot dynamics that studies the relationship between force and motion. Hogan first proposed the theory of impedance control in 1985 (Hogan, 1985). It has been used until now. Impedance control and admittance control are now the most important controller types that realize the simultaneous control of the robot end-effector (or joint) position and contact force. It makes the robot's flexible operation possible and ensures the safety of human co-workers to the greatest extent. In addition to safety factors, the stiffness control also relates to the robot's success rate in performing tasks, especially when it is in direct physical contact with people, objects, or the external environment, and the force is as important as the position target (Migliore et al., 2005). Figure 1 shows an interesting example problem that will arise in human–robot collaboration. Suppose we use the existing LfD framework to teach the robot a bunch of modularized and synthesized primitives. How will the robot select the appropriate skills from the skill library based on the current environment and the human co-worker's behavioral intentions and then generalize it to the correct goal? For example, after the robot acquires the ability to distribute books, can it accurately determine which stack of books to place the book on and plan a stable motion trajectory and a human-like stiffness regulation strategy? Most of the previous works divide human–robot collaboration problems into two independent parts: human action recognition and robot motion generation. This article proposes a framework based on the Probabilistic Movement Primitives (ProMPs) (Paraschos et al., 2013), which adopt a unified motion-stiffness skill expression that combines human action recognition and motion generation “organically.”

It is challenging that the robot cognition development meets our expectations, which can handle scenarios with a complexity level that a human found straightforward. A collaboration-enabled robot system will understand human behavior intent and then respond accordingly, where the human intention is partially embedded in the motion information itself. To not affect human movement, passive observation methods are commonly used to capture human movement information, such as a marker-based tracking system (Moeslund et al., 2006). However, phenomena such as occlusion, corrupted body tracking data due to the extremely unstructured environment, or computing power insufficiency may cause temporary observation loss or instability problems (Rabbi and Ullah, 2013). When two different motions quantified under the same sensor resolution scale are similar, the skill primitive similarity level increases. Moreover, the inevitable observation noise further aggravates the uncertainty of robots in identifying human action intents. These facts may eventually cause robots to generate unreliable reactions, which significantly reduces work efficiency; if such skill transfer technology is applied to robot-assisted surgery, it may even cause catastrophic danger. Hence, we aim to promote the collaboration capability in complex scenarios by seeking compliant physical interaction solutions with better decision making to further improve the existing LfD frameworks.

Using (surface) electromyography (i.e., sEMG) signals to predict human intentions and control robots is not a novel idea (Li et al., 2017; Chen et al., 2020a). Extracting the sEMG signal during natural human actions as an additional feature for skill expression, especially when interacting with robots or the environment, helps improve the resolution of human intention prediction results. We will use appropriate methods to extract useful information from a relatively high noise level and exploit it. Gomi and Osu (1998) examined the limb joint stiffness coefficients between shoulder and elbow; it shows that the stiffness is linear to the joint torque of the preceding joint. Burdet et al. (2000) developed a method to visualize the impedance change with respect to motion by introducing small positional displacement to human and measure the restoring elastic force. Yang et al. (2011) studied the human stiffness adaptation strategy using a well-designed experiment and revealed the possibility of transferring human stiffness adaptation skills to robots. Yang et al. (2018) later propose to use the stiffness trajectory estimated by sEMG signal to model dynamical movement primitives (DMPs). It successfully transfers the human-like stiffness regulation strategy from humans to a Baxter robot, and the framework was validated by designing cucumber-cutting and button-pushing experiments. Yu et al. (2019) then design a human–robot collaborative sawing system based on the sEMG-stiffness mapping to increase the efficiency and produce a smoother wood cutting section area.

In the previous work (Yang et al., 2018), the stiffness trajectory's generalization target was defined manually, which is empirical and biased. The reason behind this limitation is that the DMPs skill modeling technique does not reflect the coupling between motion and stiffness. Users have to manually tune the parameters for each separated DMPs or modify the DMPs expression by adding additional coupling terms, making DMPs representation not compact anymore. Very recently, Zeng et al. (2020) propose to use hidden semi-Markov models (HSMMs) and Gaussian mixture regression (GMR) to offer the capability of capturing the correlation between position, speed, and stiffness. This article proposes an entirely different approach, which is easier to understand and implement, and also suitable for human–robot collaboration. Our proposed framework has the following advantages and contributions:

- Compared to Yang et al. (2018), our framework naturally extracts the coupling between position and stiffness, which is not artificially defined with bias.
- It triggers accurately generalized robot action by observing (incomplete) human data, which is more intuitive and natural, and considers human-in-the-loop.
- We exploit sEMG-stiffness mapping for collaboration tasks so that variable stiffness regulation is achieved. The action generalization becomes more robust to the observation noise from the motion tracking system.
- Using a mixture model to learn multiple non-linear correlated skill primitives, which not only increases the diversity of the skill library but also reduces the human effort in the skill demonstration phase since similar actions are automatically sorted out.

The remaining sections of this article are structured as follows. In section 2, we introduce the basic foreknowledge about ProMP and the other methods like sEMG-stiffness mapping, ProMP modulation, and mixture model, which help to build the proposed framework. Section 3 introduce the setup of three experiments to verify the performance. And the results and discussions can be found in section 4. We list the future works in section 5 and make a final conclusion in section 6.

## 2. Materials and Methods

There are plenty of ways to express skills. Modeling a skill means synthesizing the pattern of variation for each degree of freedom of various modalities involved at the trajectory level and representing them in a more compact and utilizable way. When using different mathematical tools to promote skills modeling, each expression model naturally incorporates the tool's capabilities. Thus, the corresponding tool limitations would also apply, which provide each skill modeling technique its own usage, functionalities, and possibilities. Generally, methods of skill modeling usually fall into one of two categories, dynamical system based or probabilistic approach based.

DMPs as the most well-known dynamical system-based modeling approach first officially proposed in 2003 (Schaal et al., 2003), and the procedure was then modified and improved by numerous researchers (Ijspeert et al., 2013; Wang et al., 2016; Ugur and Girgin, 2020). DMPs earn benefits from the robust and converge-definite characteristics of the second-order spring-damper system, and the flexibility of modification by using additional forcing terms in the dynamical equation as the system's variable virtual external force to encode a motion trajectory. The patterns of the trajectories are commonly encoded using Locally weighted regression (LWR) (Atkeson et al., 1997), which is a technique that well trade off the training time and non-lineararity feature comparing to other conventional regression techniques.

Unlike DMPs, which is suitable for single demonstration modeling (i.e., one-shot learning) and learning control directly, another broad category probabilistic approach is to build a statistical model of the training information obtained through multiple demonstrations (or single demonstration). Typically, the utilization of probability theory allows the system to be more flexible in generalization, hence produces more interesting results that facilitate task planning in a higher abstraction level. Gaussian mixture model (GMM) (Hersch et al., 2008) turns both temporal information and other higher dimensional spatial information into a multi-variant Gaussian distribution containing multiple models. GMR (Khansari-Zadeh and Billard, 2011) practices the basic probability distribution operations in probability theory. The conditional probability of the Gaussian distribution and the superposition of the distribution are performed in turn to reproduce or generalize the skill from a trained GMM.

The uniqueness of DMP is not using LWR to learn weights for a bunch of radial basis functions but is the stability induced by the second-order dynamical equation. The learning speed of LWR becomes slower when the data becomes more and larger in size. Hence, to retain the advantages of using dynamical equations and speed-up, GMM–GMR can replace LWR (Calinon et al., 2012). Instead, it learns the joint distribution of the forcing term and time (i.e., the phase variable, s) of each degree of freedom, and expresses it in a GMM. Compared with GMM, the time series expressed by the Markov chain. The Markov chain encapsulates GMM or single multivariant-Gaussian in each state node and considers the transition probability between each state node. The hidden Markov model (HMM) and HSMM attach hidden variables and observation probabilities to the Markov chain (Zeestraten et al., 2016). The duration of each state of HMM is implicitly encoded in the self-transition probability, while HSMM uses a duration probability to explicitly represent. Nevertheless, these kinds of implementations based on the Expectation–Maximization (EM) algorithm (Chernova and Veloso, 2007) may encounter local optima problems, especially when the data dimension is very high, or when the demonstration data are non-linearly correlated. But that would not be a severe problem since skill training can always be done offline.

Gaussian process regression (GPR) (Forte et al., 2012) is a very generic, powerful yet brute-force probabilistic modeling method, which utilizes mean plus noises to represent a high-dimensional function. Although this method captures the coupling relationship between each degree of freedom by a very large covariance Gram matrix and generalizes the skill base on the conditional probability of the given observation value, it is prone to the temporal/spatial variability during demonstrations. Hence, it requires more demonstrations to obtain a smooth trajectory. ProMPs was formally proposed by Paraschos et al. (2013) in 2013. It is a model that combines the ideas of DMPs–LWR and the probabilistic approaches. It interprets the high-dimensional trajectory using weighted basis functions and computes the Gaussian process regression model in the weight space. This idea of using probabilistic methods in a more abstracted space of the trajectory makes the production of generalized trajectories more flexible, and the expression structure is more compact.

Most of the existing human–robot skill transfer frameworks still relying on humans to choose appropriate skill primitive among the learned primitive library, and pre-defined a generalization target. Calinon et al. (2007) propose to couple the robot with the environment (e.g., using robot–object relative position) directly and train the skill model, thereby avoiding the problem of manual selection of generalization targets. Mülling et al. (2013) put forward the concept of query for generalization and propose a Mixture of Motor Primitive (MoMP). For tasks like robot table tennis or other difficult tasks, a robot may need to switch between/combine different motion styles to complete. MoMP establishes a gating network, which adapts the styles according to queries and performs superposition among each style to generalize tasks and adapt to new scenarios. Many researchers treat action recognition and skill generalization as two separated sequential procedures and use two different models; however, our work that is inspired by “query” treats two procedures as a whole and realizes generalization using ProMPs and other techniques introduced in later sections.

### 2.1. Probabilistic Movement Primitives

**Figure 2:**
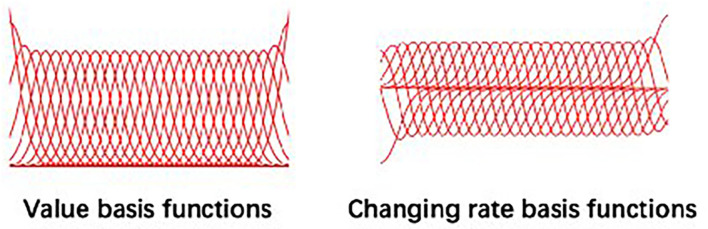
The Gaussian basis functions used in Probabilistic Movement Primitive (ProMP) model.

ProMPs encode the pattern of a high dimensional trajectory. The value of each degree of freedom (DoF) on the trajectory at time *t* is defined as *p*_*t*_. For a trajectory with a temporal length of *T* (i.e., the trajectory was sampled at number T of points), the whole trajectory is then the data points assembly ***p***_1:*T*_ = {_*p*_*t*_}*t* = 1:*T*_. The ProMP model is a probability density function that indicates the value and changing rate distribution along the single high-dimensional trajectory. The trajectory value and the changing rate at time *t* were defined in the following generic form:


(1) $$\begin{array}{l} \zeta_{t}=\left[\begin{array}{c} p_{t}\\ {ṗ}_{t} \end{array}\right]=\Phi_{t}\omega +\epsilon_{\zeta_{t}}, \end{array}$$

where $\Phi_{t}={\left[\begin{array}{cc} \psi_{t} & {\dot{\psi }}_{t} \end{array}\right]}^{T}$, which is the Gaussian basis value matrix at time *t*. It concatenates the value and changing rate of all basis function at time *t*. ***ω*** is the weighting matrix, indicating the weight of each basis function. **Φ**_*t*_ and **ω** are of the dimensionality of ℝ^2×*K*^ and ℝ^*K*×1^, respectively, where *K* is the number of basis functions pre-determined by the user. $\epsilon_{\zeta_{t}}∽N\left(0,\Sigma_{\zeta_{t}}\right)$ represents the Gaussian noise at time *t* that embraces all the possibilities of executing this trajectory in the form of a covariance, $\Sigma_{\zeta_{t}}\in {\mathbb{R}}^{2\times 2}$. This paper assuming the most common basis type—Gaussian basis—was used to model non-periodic skills as shown in Figure 2; other basis like Fourier basis and Bezier curve basis are also applicable.


(2) $$\begin{array}{l} p\left(\zeta_{1:T}|\omega \right)=\underset{t}{\prod }N\left(\zeta_{t};\Phi_{t}\omega ,\Sigma_{\zeta_{t}}\right) \end{array}$$

Equation (2) describes the probability of a trajectory ***p***_1:*T*_ conditioned on certain weighting matrix ***ω***, which is the product of *p*(***ζ***_*t*_|***ω***) from time 1 to *T*. Similar to DMPs that utilizes a temporal scaling factor and a phase variable to control the skill execution rate and represent skill completion status (Ijspeert et al., 2013), the vanilla ProMP model also uses an arbitrary monotonically increasing function *s*(*t*) as a phase variable, which interprets movement completion status and decouples movement from time (Paraschos et al., 2018). The focus of this article is not on the changes in skill execution speed or temporal modulation, hence a linear time-phase mapping [i.e., *s*(*t*) = *t*/*T*] was adopted. To simplify it, we use (·)_*t*_ to represent a variable at phase *s*(*t*).

The term “Probability” in the ProMP model originated from the fact that it relies on statistics of multiple demonstrations to improve other movement primitives like DMPs that do not model the correlation between values, rates, and time. It condenses useful information from the raw data and shrinks the data structure to output a more compact form as a single skill primitive representation. The full training set of *N* demonstrations is defined as $\zeta_{1:T}^{1:N}={\left[\begin{array}{ccc} \zeta_{1:T}^{1} & \ldots & \zeta_{1:T}^{N} \end{array}\right]}^{T}$, where $\zeta_{1:T}^{\left(n\right)}$ indicates the whole trajectory of the *n*-th demonstration. Hence, we are expected to learn a series of weights $\omega^{1:N}={\left[\begin{array}{ccc} \omega^{1} & \ldots & \omega^{N} \end{array}\right]}^{T}$for all demonstrations, where ***ω***^(*n*)^ would be the weights learned using the *n*th demonstration.

**Figure 3:**
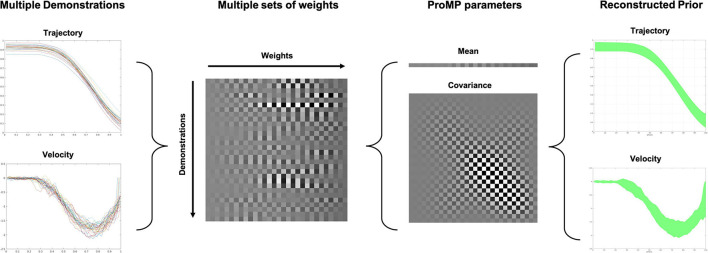
The rationale of Probabilistic Movement Primitive (ProMP) skill modeling approach. The multiple demonstrations are encoded into a number of weights. Then the Gaussian process regression is applied in the weight space, which further encoded the skill. The distribution of the trajectories can be recovered using Equation (3). The grayscale in the chessboard-like representation of the ProMP parameters indicates the normalized value for each basis. The white block means a high value, whereas the black block means low.

The essence of the original ProMP is to create a distribution over all the possible weights, so that ω∽N(ω;μ,Σ), where ***μ*** = *E*(***ω***^1:*N*^) ∈ ℝ^*K*×1^ and **Σ** = *Cov*(***ω***^1:*N*^) ∈ ℝ^*K*×*K*^. The probability of seeing the whole trajectory is computed by


(3) $$\begin{array}{l} p(\zeta_{1:T};\mu_{\zeta }^{1:T},\Sigma_{\zeta }^{1:T})=\int N(\zeta_{1:T}|\Phi_{1:T}\omega ,\Sigma_{\zeta })N(\omega ;\mu ,\Sigma )d\omega \\ \;=N(\zeta_{1:T};\Phi_{1:T}\mu ,\Phi_{1:T}\Sigma \Phi_{1:T}^{T}\\ \;+\Sigma_{\zeta }), \end{array}$$

where $\mu_{\zeta }^{1:T}\in {\mathbb{R}}^{T\times 2}$ and $\Sigma_{\zeta }^{1:T}\in {\mathbb{R}}^{T\times T}$ are the mean and covariance over the whole trajectory, representing in the trajectory space. $\Phi_{1:T}\in {\mathbb{R}}^{\left(T\times 2\right)\times K}$ is the concatenation of basis function values for all the basis and at all the time points. The reason that a number 2 exist is that it contains both trajectories of position and velocity. In summary, the ProMP model encodes the trajectory into the weighting space and the trajectories can be reconstructed based on (3). The learning of the weights is actually a least-square problem, which could be solved using Moore-Penrose inverse that projects the trajectory from the original space to a weighting space (Calinon, 2020). Figure 3 clearly demonstrates the rational of ProMP skill modeling approach.

### 2.2. Extraction of the Stiffness Adaptation Strategy Using sEMG

**Figure 4:**
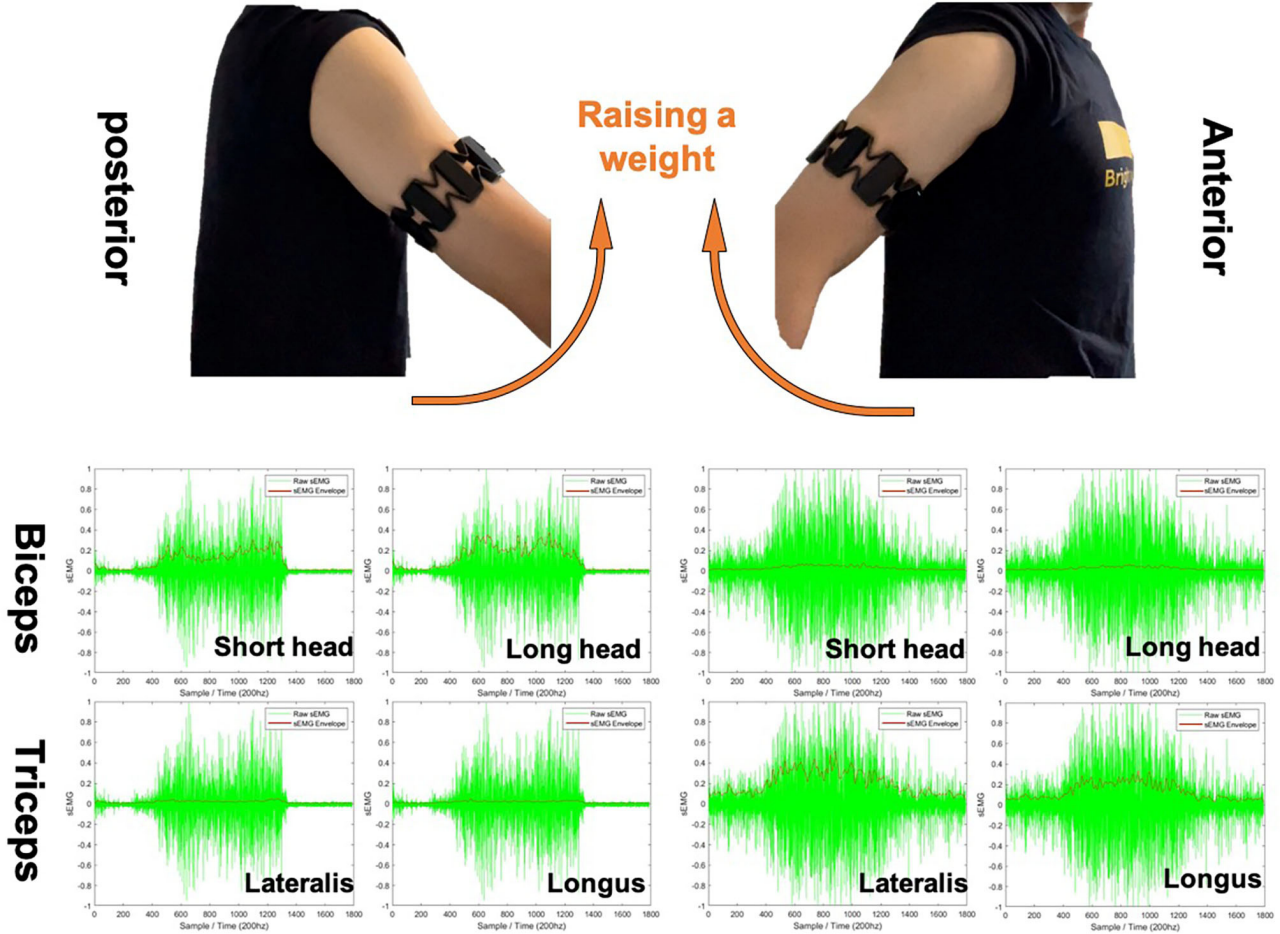
Typical example of surface electromyography (sEMG) envelope computing used in this paper based on Butterworth filter with a cut-off frequency of 5 Hz. Four sEMG channels are taken into account that cover the antagonist muscle pair biceps and triceps. The green signals are the raw sEMG signal, and the red line are the computed envelopes. Sampling interval is 10 ms.

Here, we try to explain the necessity of sEMG signals to take part in our proposed framework from two perspectives. Then the signal handling processes are introduced in this section. First, the sEMG signal is used to estimate the human arm endpoint stiffness. As our previous work demonstrates, transferring human-like stiffness adaptation strategies from human to the robot end-effector or each joint will considerably prompt the success rate of tasks requiring both force control and position control (Huang et al., 2018; Chen et al., 2020b). Second, sEMG, as the additional signal being used to produce human side stiffness indication, increases the Shannon capacity of the “communication channel” between two different agents, assuming the proposed framework has good data encoding and decoding techniques (i.e., skill modeling as encoding and action recognition as decoding). To put it simply, the positive effect of the increased number of features for skill encoding is that the robot can recognize and produce more types of actions. The sEMG signal pre-processing would be straightforward, aiming to get a fitly smooth sEMG envelope for each channel. The signal is detrended to prevent any unwanted effects like sensor drift. Then, the global mean is subtracted to remove any possible offset. After then, a certain low-pass filter could be applied based on the choice of users. Filters can be chosen from linear/root-mean-square (RMS) moving average, Butterworth filter, and any other filters with a low-pass profile. To clarify, the choice of filtering techniques with their parameter settings will certainly influence the results. The users will have to choose their own filter types depending on the choice of sEMG signal collecting device. All of the sEMG envelope results generated in this paper were based on a low-pass Butterworth filter with a cut-off frequency at 5 Hz. Algorithm 1 demonstrates the procedure of finding the envelope *a* for a single channel. Figure 4 demonstrates the typical results of a weight raising motion. The left and right images show the results of raw sEMG signals and computed envelopes while raising a weight to the posterior and anterior of body, respectively. It was easy to visualize that the muscles worked with co-activation, and the antagonistic muscles act in a opposite way to produce opposite functionalities.

**Algorithm 1:** sEMG Enveloping

**Require**: *EMG*_*raw*_; Sampling frequency *f*_*s*_; Cut-off frequency *f*_*co*_; Filter order *O*; Window length *T*_*w*_; Number of points within window *N*_*w*_
**Procedure**:
*EMG*_*detrend*_ ← *detrend*(*EMG*_*raw*_)
*EMG*_*rectified*_ ← *EMG*_*detrend*_ − *mean*(*EMG*_*detrend*_)
**if** Linear Moving Average **then**
$a\left(t\right)=\frac{1}{N_{w}}\overset{t+\frac{T_{w}}{2}}{\underset{t-\frac{T_{w}}{2}}{\sum }}\left(\left|EMG_{rectified}\left(t\right)\right|\right)$
**else if** RMS Moving Average **then**
$a\left(t\right)=\sqrt{\frac{1}{N_{w}}\overset{t+\frac{T_{w}}{2}}{\underset{t-\frac{T_{w}}{2}}{\sum }}{\left(EMG_{rectified}\left(t\right)\right)}^{2}}$
**else if** Butterworth Filter **then**
*a* = *Butterworth*(*f*_*s*_, *f*_*co*_, *O*, |*EMG*_*rectified*_|)
**end if**
**return** *a*
**End Procedure**

Because of the effect of muscle synergies, a number of muscles can contribute to the end-point stiffness of each arm (Ison and Artemiadis, 2014). If a number of *I* muscles are considered, the calculation of the muscle activation indicator *e* is given by


(4) $$\begin{array}{l} e\left(t\right)=\frac{1}{W}\overset{I}{\underset{i=1}{\sum }}\overset{t+\frac{W-1}{2}}{\underset{t-\frac{W-1}{2}}{\sum }}a_{\left(i\right)}\left(t\right), \end{array}$$

where *W* is the window length defined by users and *a*_(*i*)_(*t*) is the envelope value of the *i*th channel at time *t*. As indicated in Yang et al. (2017), the stiffness model can be simplified by using an antagonistic pair (i.e., biceps and triceps). In this paper, four sEMG channels that cover the Biceps (i.e., brachii short head and long head) and the triceps (i.e., triceps lateralis and longus) were used to estimated the stiffness. Equation (5) defines the system mechanical impedance model (e.g., of a human arm/robot manipulator) that interacts with the external environment.


(5) $$\begin{array}{l} F_{ext}=\Lambda \left(\ddot{x}-{\ddot{x}}_{d}\right)+D\left(\dot{x}-{\dot{x}}_{d}\right)+K_{cart}\left(x-x_{d}\right), \end{array}$$

where $F_{ext}$ is the external force; **Λ**, ***D*** and ***K***_*cart*_ are the inertia, damping, and stiffness matrices in the task space; ***x*** and ***x***_*d*_ are the real position and desired position in the task space, respectively. This equation implies that the interaction force can be controlled by adapting the stiffness matrix online. The Cartesian space stiffness is then estimated by a transformation from the joint space (Ajoudani et al., 2015), as shown in (6) and (7).


(6) $$\begin{array}{l} K_{cart}\left(e,q_{a}\right)={\left({\left(J_{a}\left(q_{a}\right)\right)}^{+}\right)}^{T}\left[K_{joint}\left(e,q_{a}\right)-G\left(q_{a}\right)\right]{\left(J_{a}\left(q_{a}\right)\right)}^{+} \end{array}$$

(7) $$\begin{array}{l} K_{joint}\left(e,q_{a}\right)=c\;{\bar{K}}_{joint} \end{array}$$

(8) $$\begin{array}{l} c=1+\frac{\lambda_{1}\left[1-exp\left(-\lambda_{2}e\right)\right]}{1+exp\left(-\lambda_{2}e\right)} \end{array}$$

***K***_*joint*_ is the stiffness in the joint space; ***q***_*a*_ is the human arm joint angles, and $J_{a}{\left(q_{a}\right)}^{+}$ is the pseudo-inverse of Jacobian matrix of the human arm; The human arm Jacobian is calculated based on the arm configuration estimated based on the IMU of the Myo armband. ***G***(***q***_*a*_) is a term that covers the effect of the external load and gravity on stiffness in the task space. The external load effect has been ignored since we could have no prior knowledge about the external interaction; however, the gravitational effect can be estimated for each arm configuration based on the estimated arm Jacobian. *c* is the muscle co-contraction index. λ_1_ and λ_2_ are the constants identified by the user that affect the shape of the results, and ${\bar{K}}_{joint}$ is the joint stiffness matrix under the minimum muscle activation. Since this paper does not focus on designing of impedance controller, so it is worth simplifying the framework to easily verify our algorithms. Here, we only consider the linear force components and ignore the rotation and torques. The identification of λ_1_, λ_2_, and ${\bar{K}}_{joint}$ should follow a rigorous procedures, which are different between different peoples and sEMG sensor setups (e.g., different sensors and different measuring positions on the arm). The readers could refer to the work of Ajoudani et al. (2015) and Clauser et al. (1969) for details and more information about ***G***, human arm Jacobian ***J***_*a*_ and the arm joint configuration. In this work, getting a reasonable variation profile of stiffness is already sufficient to conduct the experiments. Getting a very accurate absolute stiffness only from sEMG in real-time can be extremely difficult, for readers who are interested in estimating stiffness more accurately can refer to Fang et al. (2017) and other resources.

### 2.3. Coupling Between Motion and Stiffness in Human–Robot Collaboration

ProMP has numerous fruitful properties that capable of manipulating the model to extend the possibilities. The most important properties that we adopt in the proposed human–robot collaboration framework design are coupling and modulation. **Coupling** is a property that allows to encode the correlation between each DoFs of a high-dimensional trajectory, which is formalized as


(9) $$\begin{array}{l} \begin{array}{c} p\left(\zeta_{t,1:D}|\omega_{1:D}\right)=N\left(\zeta_{t,1:D};\Phi_{t,1:D}\omega_{1:D},\Sigma_{\zeta_{t},1:D}\right)=\\ N\left(\left[\begin{array}{c} \zeta_{t,1}\\ \vdots \\ \zeta_{t,D} \end{array}\right];\left[\begin{array}{ccc} \Phi_{t,1} & \ldots & 0\\ \vdots & \ddots & \vdots \\ 0 & \ldots & \Phi_{t,D} \end{array}\right]\left[\begin{array}{c} \omega_{1}^{T}\\ \vdots \\ \omega_{D}^{T} \end{array}\right],\Sigma_{\zeta_{t},1:D}\right) \end{array} \end{array}$$

where *D* is the total number of DoFs. ***ζ***_*t*, (*d*)_, **Φ**_*t*, (*d*)_, and ***ω***_*d*_ are the trajectory values and trajectory value changing rates, basis value matrix, and weight matrix of the *d*th DoF at time *t*. To find the distribution for the whole trajectory over all the time from 1 to *T*, (9) is substituted into (2) and (3) to integrate out ***ω***_1:*D*_, which yields $\mu_{\zeta ,1:D}^{1:T}$ and $\Sigma_{\zeta ,1:D}^{1:T}$ (i.e., mean and covariance of the *D*-dimensional trajectory from time 1 to *T*). The off-diagonal blocks of $\Sigma_{\zeta ,1:D}^{1:T}$ clearly show the coupling between each DoF. From the above, multiple features can be encoded into a single skill primitives, hence multiple agents can be coupled together (e.g., human and robot). Our proposed framework encodes the Cartesian positions (3 DoFs) and estimated endpoint stiffness (3 DoFs, see section 2.2) for human arm and robot manipulator simultaneously. Recall that ***K***_*cart*_ = *diag*([*K*_*cart,x*_, *K*_*cart,y*_, *K*_*cart,z*_]) is the Cartesian space stiffness matrix, where the diagonal terms are the stiffness constants on each axis. Therefore, six dimensions are encoded into the ProMP model for each agent (we ignored the terms in the stiffness matrix that relate to the torque and rotation, hence the full framework should involve nine DoFs for each agent). For encoding human–robot coupling, the data types are defined as


(10) $$\begin{array}{l} \zeta_{t,1:D}\leftarrow {\left[\begin{array}{cc} {\left[\zeta_{t,A}\right]}^{T} & {\left[\zeta_{t,R}\right]}^{T} \end{array}\right]}^{T}, \end{array}$$

(11) $$\begin{array}{l} \omega_{1:D}\leftarrow {\left[\begin{array}{cc} \omega_{A} & \omega_{R} \end{array}\right]}^{T}, \end{array}$$

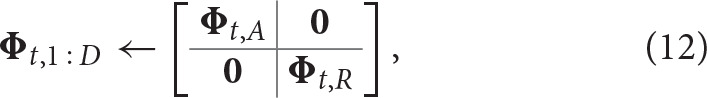

where the lower scripts (·)_*A*_ and (·)_*R*_ are used to indicate the DoFs for the human arm and the robot, respectively.

**Modulation** is the another property we used for skill generalization and adaptation (Maeda et al., 2017). Thanks to the fact that ProMP was build in a stochastic way, all the probability theories could still be applied. To implement the modulation, conditioning techniques are used. Suppose an observation $\left[{\tilde{\zeta }}_{t,a},{\tilde{\Sigma }}_{\zeta_{t},1:D}\right]$ of the human arm is performed at time *t*, where ${\tilde{\Sigma }}_{\zeta_{t},1:D}$ denotes the measurement noise. Then, the conditional distribution $p\left(\omega_{1:D}|\left[{\tilde{\zeta }}_{t,a},{\tilde{\Sigma }}_{\zeta_{t},1:D}\right]\right)$ will update the model by “slicing” on *p*(***ω***_1:*D*_). The observation can be performed at a single time or multiple times, and can be performed for either a single DoF or any subset of all the DoFs. For each time that the observation happens, the conditional distribution of the weight will be updated recursively according to


(13) $$\begin{array}{l} \Phi_{t,1:D}^{obs}\leftarrow O_{t}\Phi_{t,1:D} \end{array}$$

(14) $$\begin{array}{l} {\hat{\mu }}_{1:D}=\mu_{1:D}+\frac{\Sigma_{1:D}{\left(\Phi_{t,1:D}^{obs}\right)}^{T}\left({\tilde{\zeta }}_{t,a}-\left(\Phi_{t,1:D}^{obs}\right)\mu_{1:D}\right)}{{\tilde{\Sigma }}_{\zeta_{t},1:D}+\left(\Phi_{t,1:D}^{obs}\right)\Sigma_{1:D}{\left(\Phi_{t,1:D}^{obs}\right)}^{T}} \end{array}$$

(15) $$\begin{array}{l} {\hat{\Sigma }}_{1:D}=\Sigma_{1:D}-\frac{\Sigma_{1:D}{\left(\Phi_{t,1:D}^{obs}\right)}^{T}\left(\Phi_{t,1:D}^{obs}\right)\Sigma_{1:D}}{{\tilde{\Sigma }}_{\zeta_{t},1:D}+\left(\Phi_{t,1:D}^{obs}\right)\Sigma_{1:D}{\left(\Phi_{t,1:D}^{obs}\right)}^{T}}, \end{array}$$

where ${\hat{\mu }}_{1:D}$ and ${\hat{\Sigma }}_{1:D}$ are the updated weight space mean and covariance of the modulated model, and ***O***_*t*_ is an observation matrix that comprises identity matrices ***I*** ∈ ℝ^2×2^ and zero matrices **0** ∈ ℝ^2×2^, indicating which DoF(s) is(are) observed at time t (when only observing the position, the value one at the second diagonal entry of ***I*** is replaced with zero). ***O***_*t*_ is then officially defined as


(16) $$\begin{array}{l} O_{t}\leftarrow {\left[\begin{array}{ccc} {\left[I\;or\;0\right]}_{1} & \ldots & {\left[I\;or\;0\right]}_{D} \end{array}\right]}^{T} \end{array}$$

**Figure 5:**
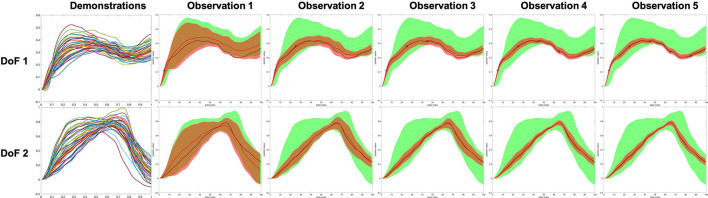
A two degree of freedoms (DoFs) example showing how the model being modulated while maintaining the coupling between DoFs. The shade indicates the 2-σ confidence of the value. The green and red shades are representing the prior and modulated inference, respectively. The red curve and black curve are the mean of the inference and the demonstrated ground truth, respectively. Five points of the DoF 1 were observed, and the algorithm updates (modulates) both DoF 1 and DoF 2 iteratively.

Using the updated weight space mean and covariance in (14) and (15), the task space mean $\mu_{\zeta ,1:D}^{1:T}$ and covariance $\Sigma_{\zeta ,1:D}^{1:T}$ are then reconstructed using (9), (2), and (3). Consequently, all the human/robot DoFs can be inferred/modulated by conditioning on observation; hence, a natural robot action generalization process is achieved. Figure 5 shows a two DoFs example that clearly shows how model being modulated when considering the coupling between two DoFs.

### 2.4. Learning and Inference of Multiple Skill Primitives

**Figure 6:**
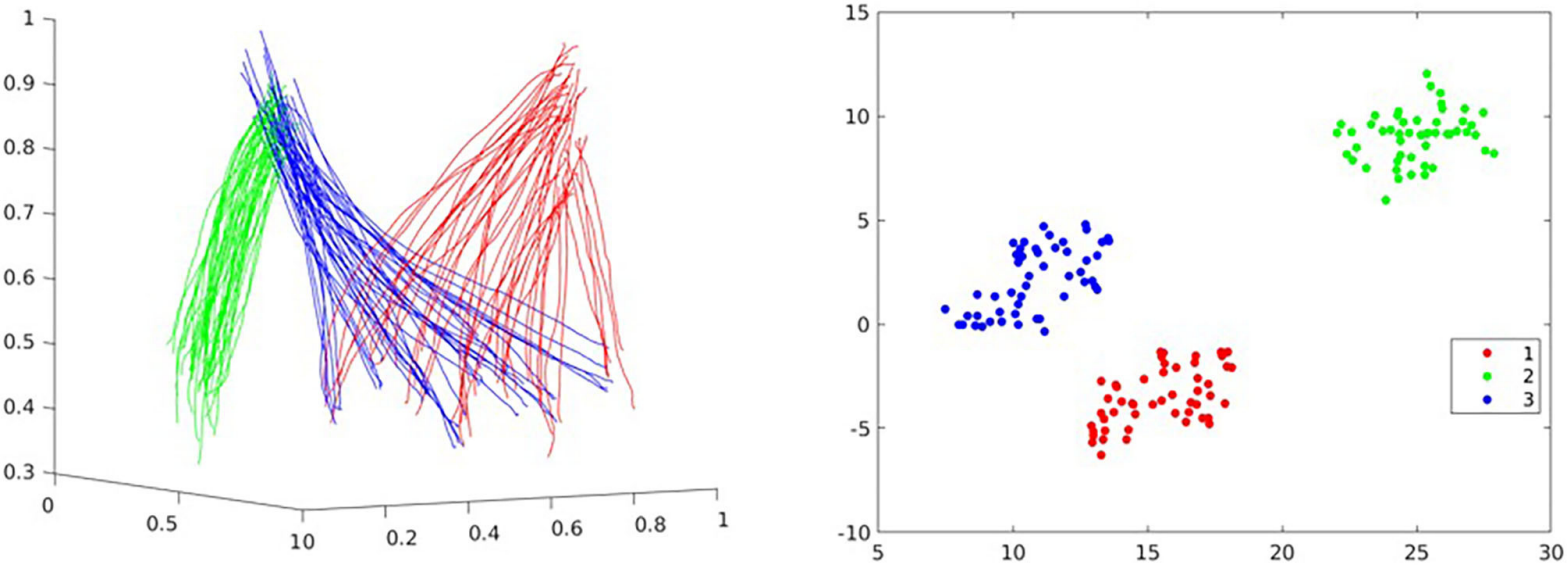
Demonstrations for a pick-and-place task and its weights data visualization using t-distributed stochastic neighbor embedding (t-SNE): reach (red), pick (blue), and place (green).

The above sections consider only learning a single skill (assuming that the demonstrations for that skill are linearly correlated). In a real human–robot collaboration scenario, we need to consider making the robot possess many general skills and infer human intentions by observing a small amount of information and selecting the appropriate primitive in the skill library for generalization. The modeling of a single ProMP actually assumes that the weights of the Gaussian basis functions in a unified skill conform to a single modal multi-variant Gaussian distribution. As shown in Figure 6, we have obtained a set of pick-and-place demonstration data. In that figure, red represents the reach action, blue represents the pick action, and the green represents the place action. We use t-distributed stochastic neighbor embedding (t-SNE) (Van der Maaten and Hinton, 2008) to visualize all the sets of learned weights. It is clearly shown that the three behaviors are filed into three categories. This encourages us to model non-linear correlated skills using a GMM.

Assuming that the robot is expected to learn Q types of collaborative skills, our learning objective is a GMM distribution with Q local modals $p\left(\omega_{1:D};\pi^{\left(q\right)},\mu_{1:D}^{\left(q\right)},\Sigma_{1:D}^{\left(q\right)}\right)$, where *q* ∈ [1, *Q*]. **π**^(*q*)^ is the prior of the modal choice *q*. $\mu_{1:D}^{\left(q\right)}$ and $\Sigma_{1:D}^{\left(q\right)}$ are the mean and covariance of weights of the *q*-th local Gaussian modal. Hence, the GMM of weights is defined as


(17) $$\begin{array}{l} p\left(\omega_{1:D}\right)=\underset{q}{\sum }p\left(q\right)p\left(\omega_{1:D}|q\right)=\underset{q}{\sum }\pi^{\left(q\right)}p\left(\omega_{1:D}|\mu_{1:D}^{\left(q\right)},\Sigma_{1:D}^{\left(q\right)}\right) \end{array}$$

There are two approaches to learn the GMM parameters. The learning under human supervision would be straightforward. While in the demonstration process, human knows exactly about the category of the skill. Hence, for each demonstration, the label *q* is given. Hence, the rest of the work would be just training each local Gaussian modal individually, and the prior ***π***^(*q*)^ can be calculated based on the number of demonstrations in each category *q*. To reduce human effort, unsupervised learning methods like the EM algorithm can also be implemented. However, each set of weights of a ProMP is a very high-dimensional vector, typically more than a hundred dimensions; clustering algorithms can still result in local optima. Therefore, a validation procedure would be vital. Moreover, it is worth reminding that the log probability is always adopted to reduce the chance of encountering underflow issues since computing likelihood that involves very high-dimensional vectors often leads to an extremely small number. Using results of a K-mean algorithm as initialization of the EM algorithm would also reduce the chance of getting an error result.

After the GMM of weights is obtained, Bayes inference can be applied to recognize human action and get the best choice of label *q* = *q*_*est*_ based on the observation $\left[{\tilde{\zeta }}_{t,a},{\tilde{\Sigma }}_{\zeta_{t},1:D}\right]$, where *q*_*est*_ is the estimated action label.


(18) $$\begin{array}{l} q_{est}=\arg\underset{q}{\max}p\left(q|{\tilde{\zeta }}_{t,a}\right)=\arg\underset{q}{\max}p\left({\tilde{\zeta }}_{t,a}|q\right)p\left(q\right) \end{array}$$

(19) $$\begin{array}{l} p({\bar{\zeta }}_{t,a}|q)=\int p({\bar{\zeta }}_{t,a}|\Phi_{t,1:D}^{obs}\omega ,{\bar{\Sigma }}_{\zeta_{t},1:D})p(\omega |\mu_{1:D}^{\left(q\right)},\Sigma_{1:D}^{\left(q\right)})\\ \;=N\left({\bar{\zeta }}_{t,a};\Phi_{t,1:D}^{obs}\mu_{1:D}^{\left(q\right)},\Phi_{t,1:D}^{obs}\Sigma_{1:D}^{\left(q\right)}{\left(\Phi_{t,1:D}^{obs}\right)}^{T}+{\bar{\Sigma }}_{\zeta_{t},1:D}\right), \end{array}$$

The inference of *q*_*est*_ considers a series of observations at arbitrary times. Typically, more the observations used, the more confident about the inference results. Finally, the model was modulated using all the observations and the parameters of the local Gaussian modal $\mu_{1:D}^{\left(q_{est}\right)}$ and $\Sigma_{1:D}^{\left(q_{est}\right)}$ based on Equations (14) and (15).

## 3. Experiments

We design a series of experiments to verify the practicability of the proposed framework. The hardware used in the experiments includes Myo Armband, Leap motion, and PC. Myo armband is a wireless sEMG signal monitoring device, which has a maximum sampling rate *f*_*s*_ of 200 Hz. Myo has eight sEMG channels that designed to be fixed on the human arm. Leap motion is a hand and finger tracking device, which utilizes monochromatic IR cameras and infrared LEDs to operate. As shown in **Figure 8**, in the experiment, the armband was worn on the right upper arm to measure the sEMG data of the antagonistic muscles (i.e., biceps and triceps) of the human demonstrator and estimate the stiffness of the arm endpoint according to the method introduced in section 2.2. Hence, in the Equation (4), *I* = 4. We adopted a Butterworth filter for sEMG signal processing since the cut-off frequency can be controlled, where *O* = 3 and *f*_*co*_ = 5*Hz*. Leap motion was placed on a flat surface, and the hand was required to move in the cone shape workspace above the Leap motion camera.

**Figure 7:**
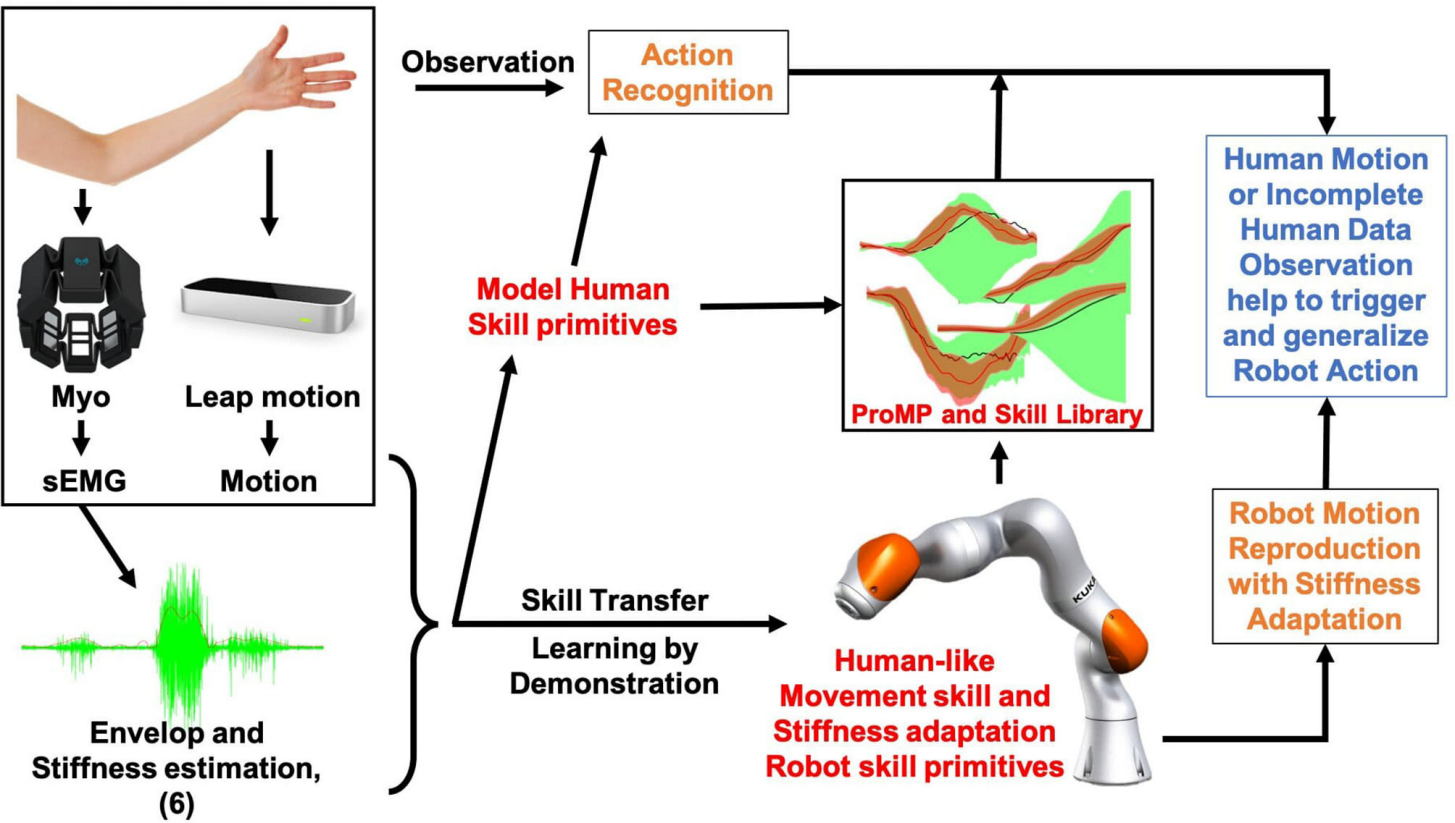
Proposed motion with stiffness adaptation skill transfer and generalization framework.

The rationale and basic workflow of the proposed framework is shown in Figure 7 intuitively. In order to transfer the human-like stiffness adaptation skills, the human arm endpoint stiffness adaptation trajectories estimated using sEMG signal are added as additional features to the skill model. In order to make the robot autonomously finds the generalization target of the desired stiffness based on different generalized motions, we model the stiffness feature through ProMP innovatively. Humans can then naturally impart the coupling between stiffness and motion to robots during demonstrations. Further, to achieve better collaboration between humans and robots, we suggest combining our previous research outcomes on stiffness adaptation skill with the benefits of ProMP in modulation. We model the human skills and robot skills simultaneously to establish coupling so that robots can generalize appropriate actions and collaborate with humans even when they have incomplete observations of human signals.

### 3.1. Hand-Over Experiment

**Figure 8:**
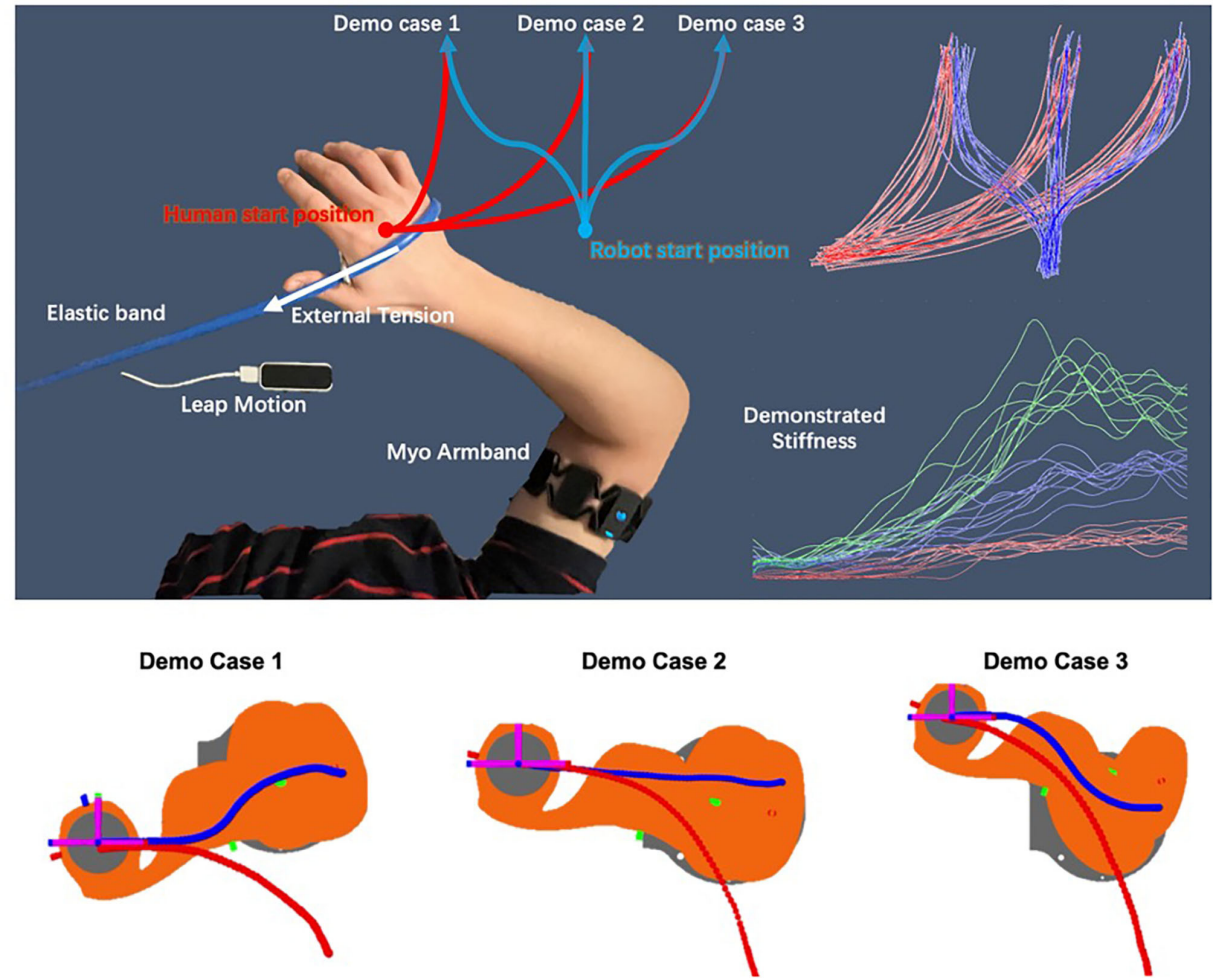
Schematic plot of the hand-over task and the demonstrations. A virtual teleoperation is conducted for the robot side demonstrations in simulation.

To verify that our framework can naturally encode the coupling of stiffness and motion, a hand-over experiment scenario (see Figure 8) was designed. In the experiment, people pulls one end of the elastic band and quickly, stably and naturally move along the given red trajectories on the x-y plane in 2 s, the captured hand motion and estimated stiffness are recorded to learn motion primitives for the human side. The other end of the elastic band is fixed on a pile. During the movement, the demonstrator's arm experiences three stages: no external tension, external tension occurs, and increasing external tension. To maintain a high trajectory tracking accuracy in the presence of external forces within 2 s, the demonstrator will perform with an adaptive stiffness strategy.

To demonstrate the robot skills, a virtual teleoperation scene in Matlab simulation was built, which use the same demonstration interface to control the robot end-effector to move along each desired blue trajectory in a simulation within 2 s. The simulated robot was controlled using a simple Cartesian positional PID controller and inverse kinematics solver with the captured human hand position as the desired position. While teleoperating the blue trajectories, the red trajectories are also replayed. For each demo case, the demonstrator is asked to perform 10 demonstrations, hence *N* = 30 in total. All the recorded data are then used to train a 12-dimensional ProMP model, where 3 DoFs for human arm position, 3 DoFs for the human arm translational stiffness, and the same DoFs apply to the robot. The number of weights in this experiment is set to be *K*=30. Using the conditioning method introduced in section 2.3, the demonstrator then shows 6 new motions to verify the framework's generalization ability and accuracy. Ideally, by only observing the last time point of the arm position trajectory, it can still infer an appropriate stiffness trajectory and an appropriate robot motion.

### 3.2. Object Matching Experiment

**Figure 9:**
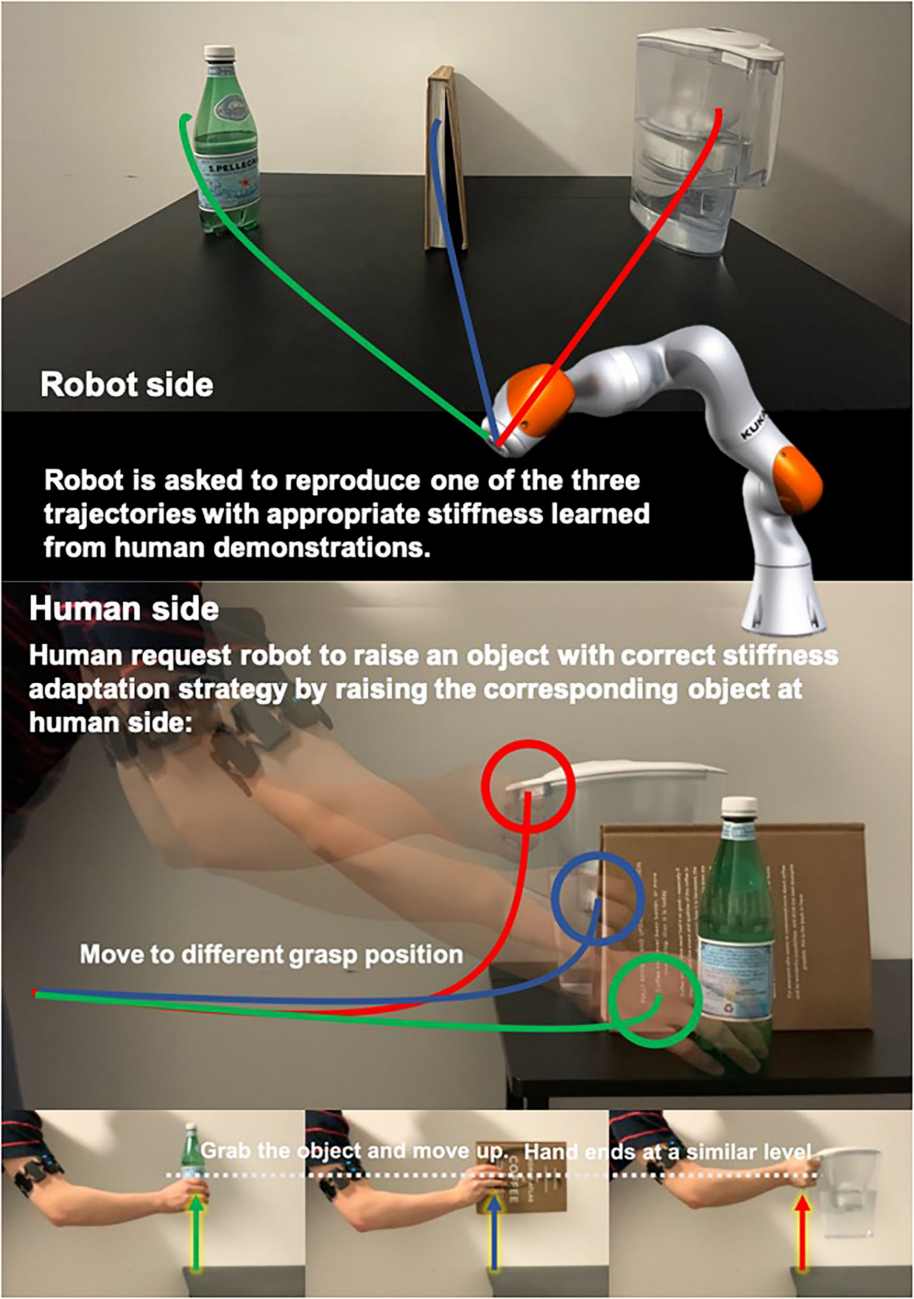
Illustration of the object matching experiment.

As shown in Figure 9, we design an object matching task to test our framework. We test our framework that has the ability to distinguish between human intents by adding stiffness information to the model when motions are similar. Using too large or small stiffness to pick up heavy object would result in motion fluctuations or pick-up failures, respectively. Hence, in this task, the demonstrator is first asked to demonstrate the pick-up skill with stiffness adaptation strategies for encoding robot side skill primitive. For each robot side trajectory, demonstrator also demonstrate the human side pick-up motion. Each object will have 10 demonstrations. The red, blue, and green lines at human side in Figure 9 illustrate the human motions. For each object, the motion trajectory will only be differed at the grasping positions since we grasp at different levels. Although the human side motions are similar, the stiffness trajectories can be significantly differed. Finally, the demonstrator pickups different objects at human side as observation to trigger modulated robot actions and see if that system performs as expected (e.g., generate motion and stiffness trajectories correctly).

### 3.3. Pick-and-Place Experiment

We use the recorded pick-and-place demonstrations, as shown in Figure 6, to verify the framework's ability to recognize human action; hence, we choose correct action label to generalize the ProMP according to observations. Three actions are considered as primitives, which are reach, pick, and place, respectively. We were assuming the object is initially located at a random position inside a circular area. Therefore, all the demonstrations of reach start at a similar position but end in different places, while the pick demonstrations start at different positions but end in a similar position. The place demonstrations should always travel along a similar trajectory. Each primitive has 50 demonstrations, thus 150 in total. All the parameter settings are the same as before, and all 150 demonstration are then used as test data to verify the framework performance in action recognition. For each demonstration, 10 equally spaced data point were chosen as the observations.

## 4. Results and Discussion

### 4.1. Hand-Over Experiment

**Figure 10:**
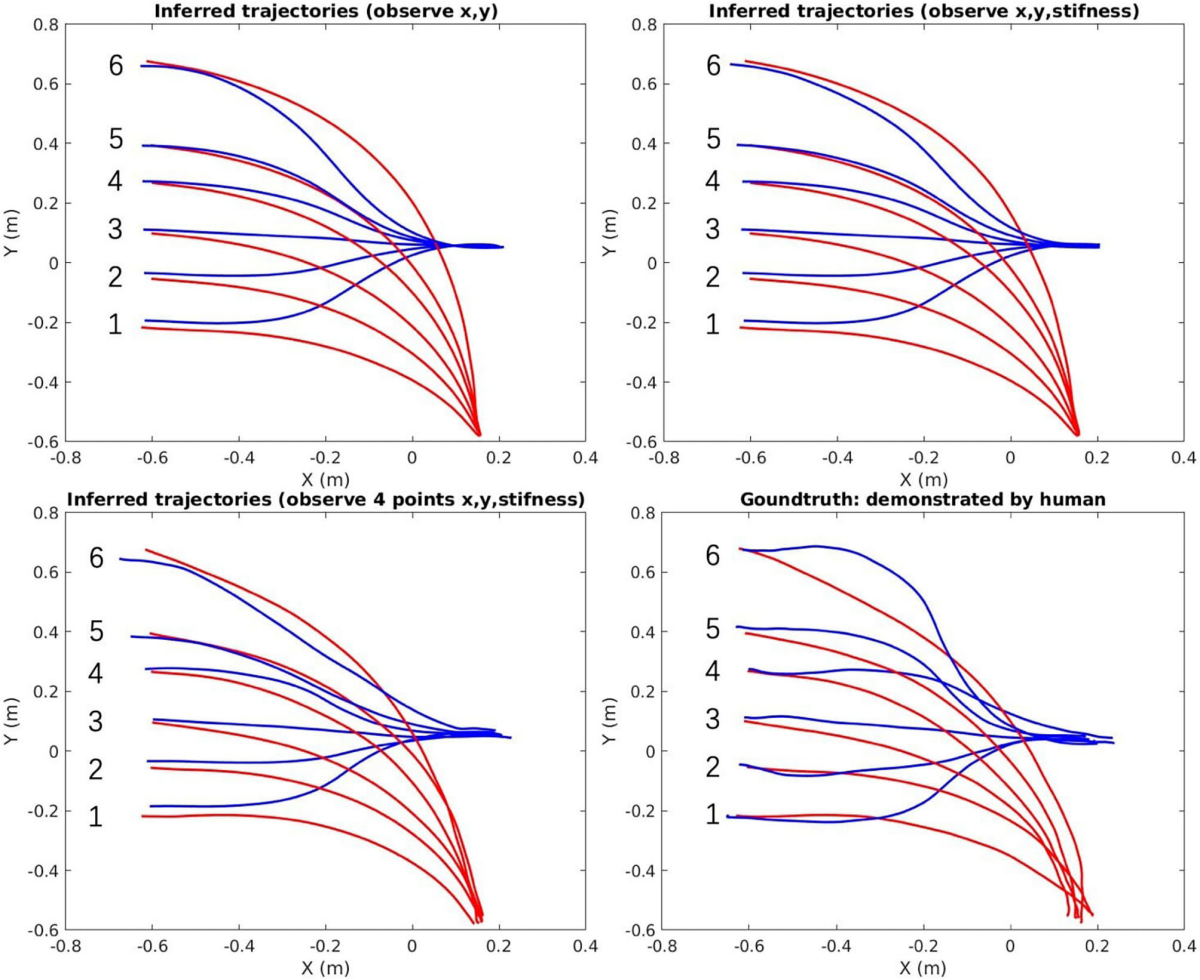
The inferred trajectories vs. the ground truth of the hand-over task [red (human arm)/blue (robot)].

**Figure 11:**
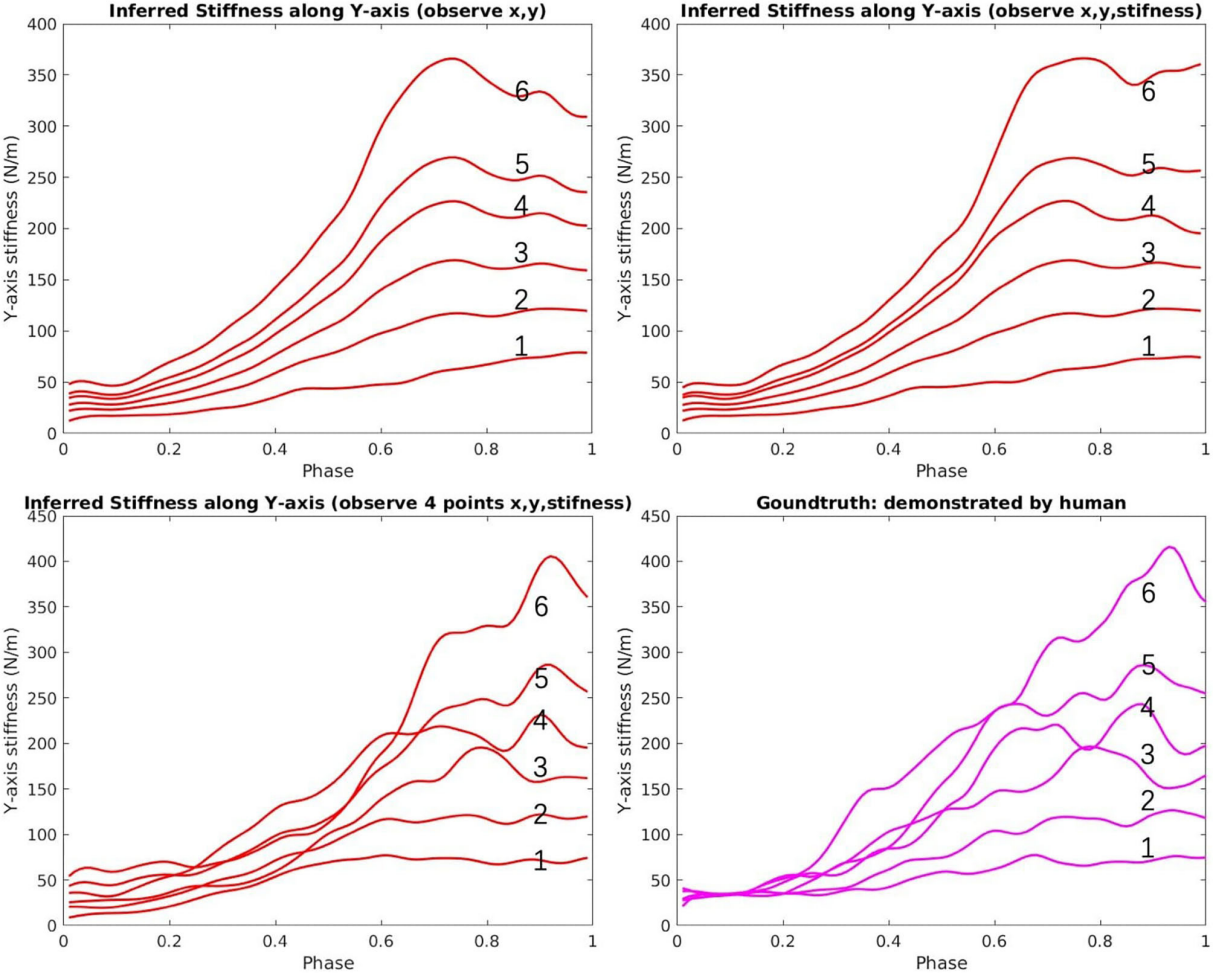
The inferred stiffness along y-axis vs. the ground truth of the hand-over task [red (inferred)/magenta (recorded ground truth)].

The top-right area in Figure 8 shows all the demonstrated trajectories for both motion and estimated stiffness *K*_*cart,y*_ along the y-axis. As shown in Figures 10, 11, the reproduced action with index 1, 3, and 5 corresponds to the demo case 1, 2, and 3 in Figure 8, respectively. The rest three reproduced actions with index 2, 4, and 6 are the novel cases to test the generalization accuracy. Three trajectory inference results are shown in Figure 8. The upper-left one shows the result that only observing human arm x-y positions at time 2 s. The upper-right one shows the result that observes human arm x-y positions and estimated stiffness at time 2 s. The lower-left one shows the result that observes human arm x-y positions and estimated stiffness at time 1.4, 1.6, 1.8, and 2 s. Comparing with the recorded ground truth at the lower-right, we can see that all cases can generalize reasonably well trajectories and complete the hand-over task. It can be seen that the ground truth trajectories are not ideally smooth in shape even though we call it the “Ground Truth.” That is because single demonstration always having large bias, using a larger number of demonstrations to synthesize the data will make the trajectory smooth and reliable. And that is further proved by comparing two upper results with the lower results. When robot inferring actions by conditioning on biased information, the model “believes” more about “bias,” while the upper two cases “believe” more about the synthesized skill trajectories mean, which makes them looks more reliable.

In Figure 11, the upper-left graph shows the results of inferred stiffness along the y-axis when only observing the x-y position of the arm at time 2 s. It proves that our proposed ProMP-based framework can encode coupling between motion and stiffness. The larger travel-distance along the y-axis, the larger stiffness along the y-axis is required, which is the tendency we expect. The upper-right graph shows that the stiffness trajectories were updated by observing stiffness at 2 s. The lower-left graph shows the same problems of “believing in bias”; however, that can also be utilized to generate different action stylish when there are various distinct styles among demonstrations. Overall, the framework works more ideally when (1) human DoFs are fully observable; (2) model trained by a sufficiently large number of demonstrations; (3) inferring robot actions by observing (conditioning) at a single time point (to reduce bias).

### 4.2. Object Matching Experiment

**Figure 12:**
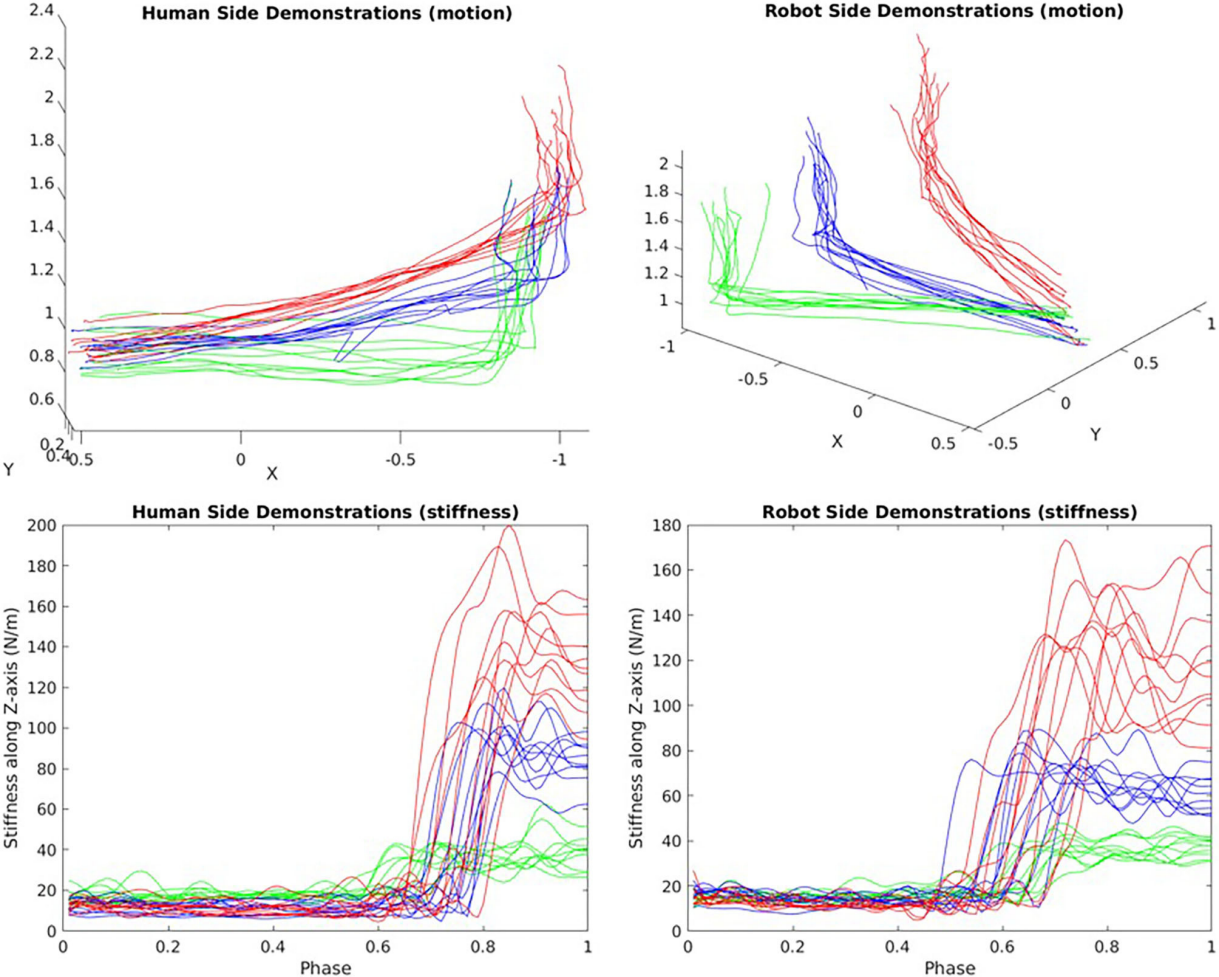
Pick-up task Demonstrations of motion and stiffness along the Z-axis (red: water filter/blue: book/green: bottle).

The human demonstrations are all shown in Figure 12. The upper-left shows that although the 3 kind of motions are distinguishable by human's inspection, it may also be vague to tell when noise exists. The lower two graphs are the demonstrated stiffness along Z-axis since that is the most interested axis when picking up objects. It can be seen that the bottle requires the least amount of stiffness to raise while the water filter requires the largest stiffness. From the graph, we see that a human use a very good stiffness control strategy. When grasping object, because the exacted weight of that object is not known, humans create an over shoot of stiffness to ensure that the task space motion is not off-track. After that, the arm will swiftly reduce the stiffness to an appropriate value so that the energy consumption is minimized.

**Figure 13:**
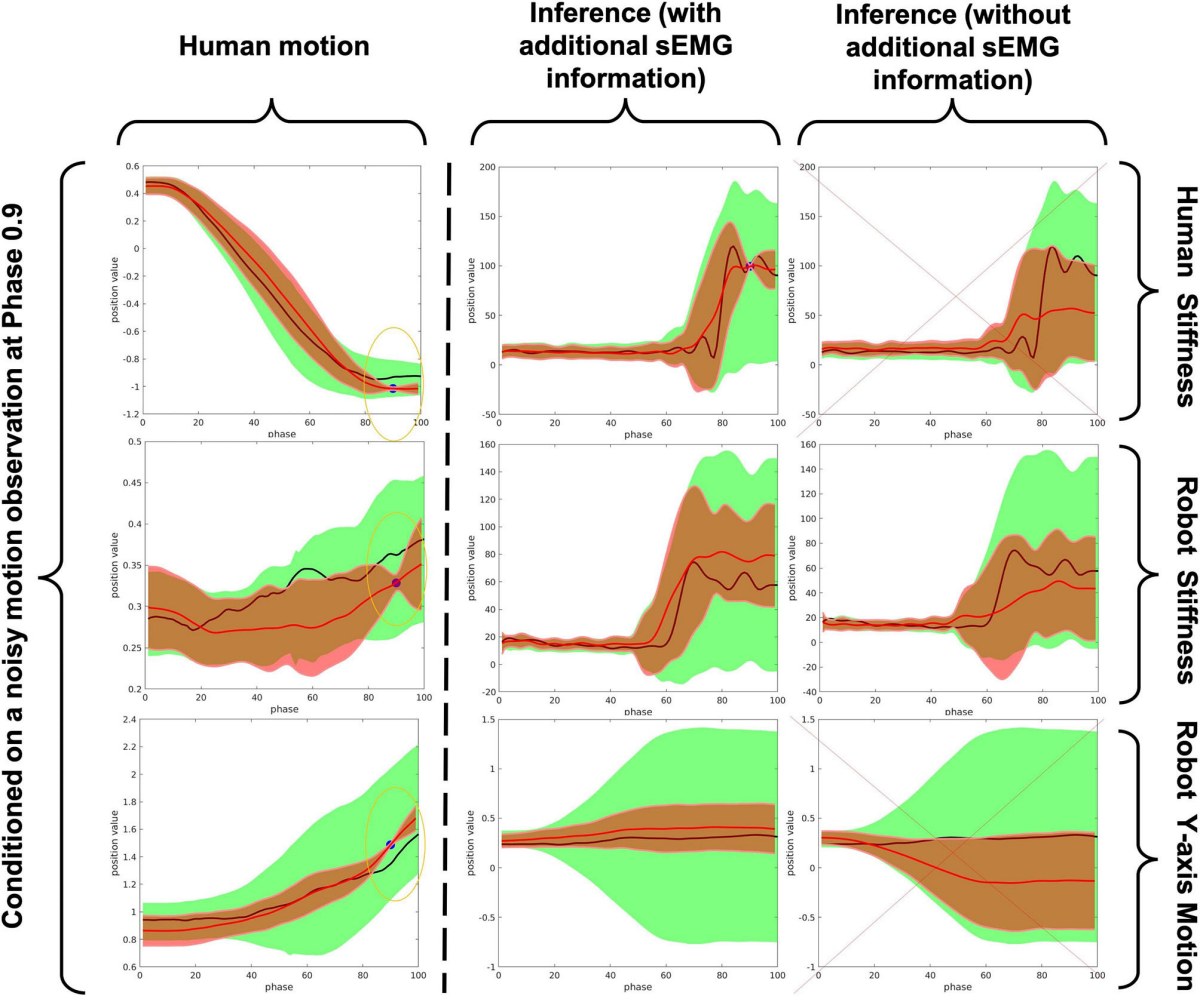
A comparison between the inference results of with and without using sEMG signal when conditioned on a motion observation with a reasonable amount of noise (red trajectory: inference of mean trajectory/black trajectory: human demonstrated ground truth/green area: 2-σ region of covariance of the pre-trained ProMP/red area: 2-σ region of covariance of the modulated ProMP/blue point: noisy observation).

Figure 13 clearly demonstrates the effectiveness of out proposed framework in distinguishing human action and generate accurate robot motion with correct amount of stiffness activation when the actions are similar and observation noise exists. The blue points is the observation; in this experiment, we adopt a random generated noise of a very reasonable amount; such amount of noise may also be the bias of human motion in each run-time (i.e., people do not always have a 100% correct motion). We clearly see that our framework works very well under the help of sEMG signal (see the middle column). This is compared to the results with no help of sEMG, where the inference process provides a absolutely incorrect human stiffness and robot motion and the robot stiffness inference is not what we expect (low stiffness activation when raising the object, this may leads to a distortion of motion trajectory during execution in the real application). Hence, we could say that the proposed framework can be used as an effective command sending interface to trigger appropriate robot actions in a very intuitive and convenient manner, which greatly reduce the effort of reprogramming.

### 4.3. Pick-and-Place Experiment

**Figure 14:**
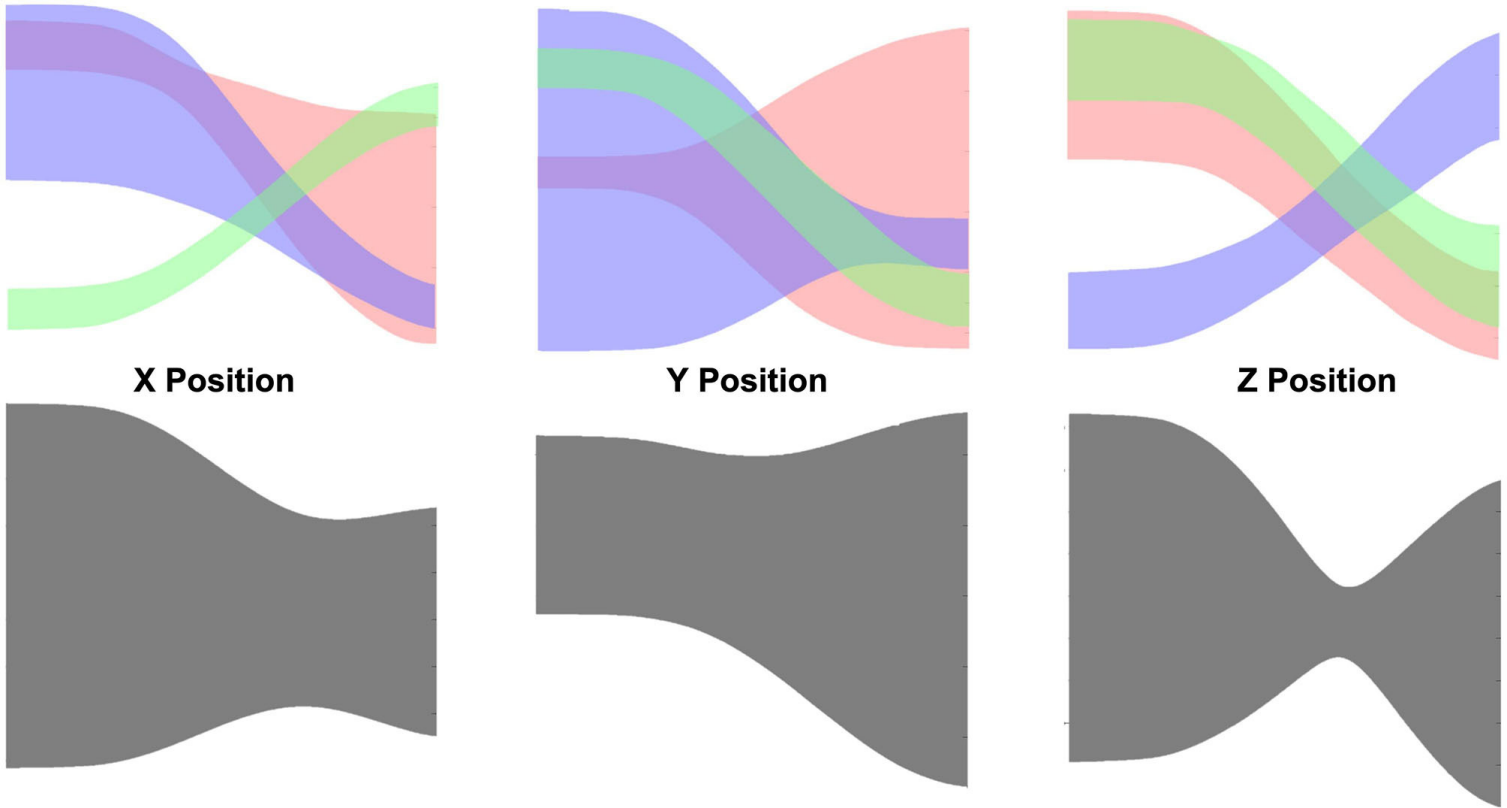
**(Top)** The learned mixture of ProMP model for pick-and-place tasks: reach (red), pick (blue), place (green). **(Bottom)** The learned ProMP model that treat non-linearly correlated actions as a single skill type.

**Figure 15:**
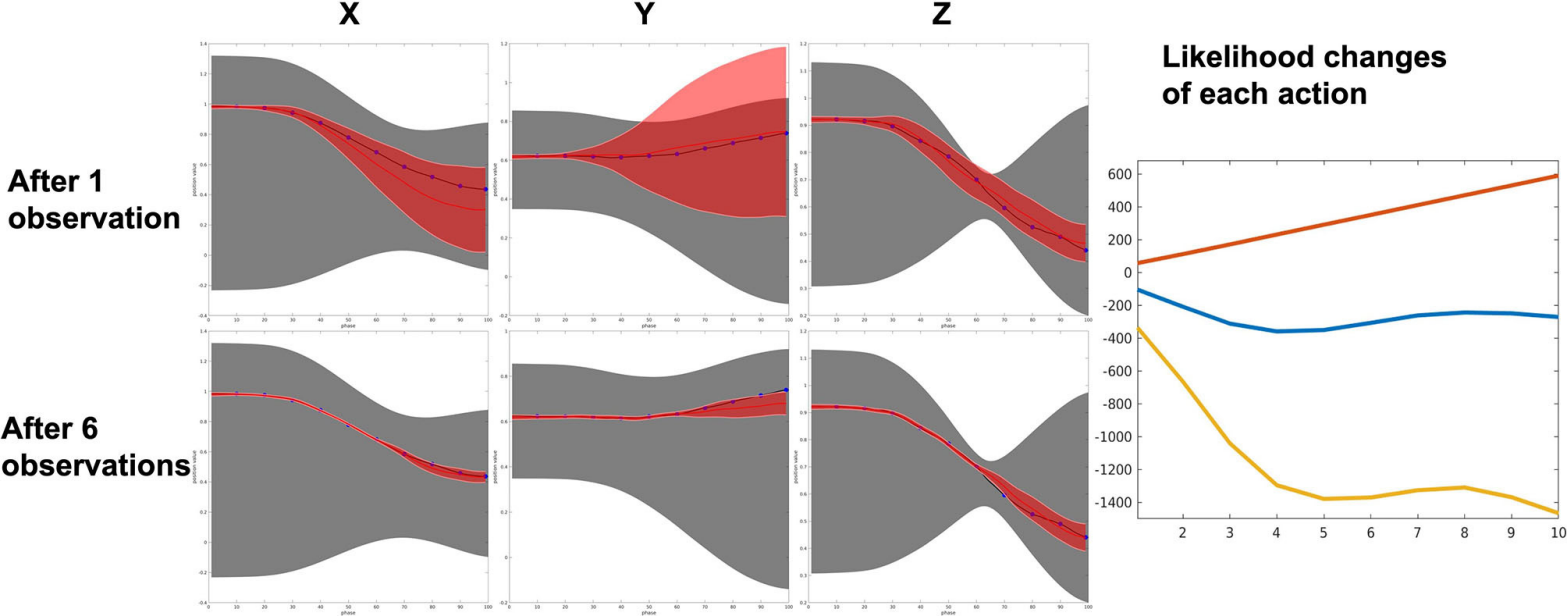
An example of reaching action recognition by 10 observation data. **(Top)** The proposed framework can recover the correct ProMP model from a mixture model. **(Bottom)** Reach action is identified and generalized based on observations. Right: Log likelihood of reach (red) is far higher than the other two actions, although overlapping of the prior make cause certain ambiguity.

The top side of Figure 14 shows the learned prior of reach (red), pick (blue), and place (green) individually. Obviously, the ProMP model learned by non-linearly correlated demonstrations (i.e., gray shade in Figure 14) cannot be used to generalize or recognize motions. All 150 tested demonstrations result in a 100% success rate. An inference of a test demonstration is considered to be succeeded if the action label is correctly inferred. An example of recognition and generalization of a reach action is shown in Figure 15. Once the action label is correctly labeled, the sum of the squared difference between the ground truth and inferred trajectory can be very low, as can be seen in Figure 15. As more and more observations coming, the log-likelihood of reach action keeps increasing with very high confidence. The overlapping of the prior (mostly occurs in the middle) does increase the likelihood of the other two actions; however, our algorithm considers the whole observation sequence from the beginning to the current time. Hence, the would be no significant ambiguity caused by overlapping.

Reducing the number of observations or increase the number of Gaussian models (i.e., skills) stored in the Gaussian mixture ProMP will introduce more inference ambiguity. However, since the proposed framework considers using stiffness profile as additional features, the inference accuracy may still keeping at a satisfying level. In our framework, the action recognition functionality is associated with the ProMP model itself since it require the prior knowledge stored in the ProMP (i.e., $\mu_{1:D}^{\left(q\right)}$, $\Sigma_{1:D}^{\left(q\right)}$). Then, the prior knowledge is modulated based on the recognized action label and all the observed information, which makes the robot be able to adapt actions to accommodate different situations.

## 5. Future Work

The action recognition functionality allows identifying the most similar actions in the learned skill library. This implies that we could find skill substitution for any coming novel action without learning and storing them into the skill library. The idea of blending in ProMP could transform one type of action to another, which means the learned skills could still be disassembled and then regrouped as a substitute for novel action. To investigate the possibility of fully automated robot operations, we can combine the ProMP-based framework proposed in this article with concepts such as skill execution sequence, environmental awareness, and affordance. Time series such as HMM can be used to model and train the robot's task-level planning ability. This article largely focuses on the theoretical study. When considering real robot control, we will encounter many foreseeable and new challenges, such as motor acceleration not reaching the desired speed, difficulties in implementing human–robot real-time collaboration, etc.

## 6. Conclusion

This paper demonstrates an adaptive stiffness human–robot skill transfer framework based on ProMP for collaborative tasks, which is very easy to understand and is effective. We discuss the importance of stiffness property in real applications and propose to use sEMG signal to estimate human arm endpoint stiffness, which can then be transferred to the robot. Moreover, the use of sEMG increases the generalization accuracy and decision-making success rate. We also illustrate why the ProMP model has benefits in building such a skill model. To prove our idea, we design experiments using the Myo armband and Leap motion, which gives results that positively support our work. We find the coupling between the adaptive stiffness strategy and motion can be encoded and transferred from humans to robots in a very intuitive manner comparing to other works. The proposed framework can be used as an intuitive interface to trigger robot action generalization via observing human action, ideal for a human–robot collaboration scenario. In the future, we will exploit the other properties of ProMP and other techniques, like skill combining and blending, mixture models to improve the flexibility of our framework further, and verify it using real robots.

## Author Contributions

YG conceptualized the framework, developed the software, designed and conducted the experiments, and wrote the paper. NW and CY supervised, reviewed, and approved the work. All authors contributed to the article and approved the submitted version.

## Funding

This work was partially supported by Engineering and Physical Sciences Research Council (EPSRC) under Grant EP/S001913.

## Conflict of Interest

The authors declare that the research was conducted in the absence of any commercial or financial relationships that could be construed as a potential conflict of interest.

## Publisher's Note

All claims expressed in this article are solely those of the authors and do not necessarily represent those of their affiliated organizations, or those of the publisher, the editors and the reviewers. Any product that may be evaluated in this article, or claim that may be made by its manufacturer, is not guaranteed or endorsed by the publisher.
